# A Web-Based Physical Activity Intervention for Spanish-Speaking Latinas: A Costs and Cost-Effectiveness Analysis

**DOI:** 10.2196/jmir.6257

**Published:** 2017-02-22

**Authors:** Britta Larsen, Bess Marcus, Dori Pekmezi, Sheri Hartman, Todd Gilmer

**Affiliations:** ^1^ University of California, San Diego Department of Family Medicine and Public Health La Jolla, CA United States; ^2^ University of Alabama at Birmingham Birmingham, AL United States

**Keywords:** cost-effectiveness, physical activity, Latinos, Latinas, Web-based interventions, health disparities

## Abstract

**Background:**

Latinas report particularly low levels of physical activity and suffer from greater rates of lifestyle-related conditions such as obesity and diabetes. Interventions are needed that can increase physical activity in this growing population in a large-scale, cost-effective manner. Web-based interventions may have potential given the increase in Internet use among Latinas and the scalability of Web-based programs.

**Objective:**

To examine the costs and cost-effectiveness of a Web-based, Spanish-language physical activity intervention for Latinas compared to a wellness contact control.

**Methods:**

Healthy adult Latina women (N=205) were recruited from the community and randomly assigned to receive a Spanish-language, Web-based, individually tailored physical activity intervention (intervention group) or were given access to a website with content on wellness topics other than physical activity (control group). Physical activity was measured using the 7-Day Physical Activity Recall interview and ActiGraph accelerometers at baseline, 6 months (ie, postintervention), and 12 months (ie, maintenance phase). Costs were estimated from a payer perspective and included all features necessary to implement the intervention in a community setting, including staff time (ie, wages, benefits, and overhead), materials, hardware, website hosting, and routine website maintenance.

**Results:**

At 6 months, the costs of running the intervention and control groups were US $17 and US $8 per person per month, respectively. These costs fell to US $12 and US $6 per person per month at 12 months, respectively. Linear interpolation showed that intervention participants increased their physical activity by 1362 total minutes at 6 months (523 minutes by accelerometer) compared to 715 minutes for control participants (186 minutes by accelerometer). At 6 months, each minute increase in physical activity for the intervention group cost US $0.08 (US $0.20 by accelerometer) compared to US $0.07 for control participants (US $0.26 by accelerometer). Incremental cost-per-minute increases associated with the intervention were US $0.08 at 6 months and US $0.04 at 12 months (US $0.16 and US $0.08 by accelerometer, respectively). Sensitivity analyses showed variations in staffing costs or intervention effectiveness yielded only modest changes in incremental costs.

**Conclusions:**

While the Web-based physical activity intervention was more expensive than the wellness control, both were quite low cost compared to face-to-face or mail-delivered interventions. Cost-effectiveness ranged markedly based on physical activity measure and was similar between the two conditions. Overall, the Web-based intervention was effective and low cost, suggesting a promising channel for increasing physical activity on a large scale in this at-risk population.

**ClinicalTrial:**

Clinicaltrials.gov NCT01834287; https://clinicaltrials.gov/ct2/show/NCT01834287 (Archived by WebCite at http://www.webcitation.org/6nyjX9Jrh)

## Introduction

Physical activity is associated with a lower risk of heart disease, stroke, type 2 diabetes, depression, and some cancers [1]; however, only about 1 in 5 (21%) US adults meet the 2008 Federal Physical Activity Guidelines [2]. Recent analyses estimate that US $131 billion (95% CI $91 billion-$172 billion) of health care expenditures per year are associated with inadequate levels of physical activity. Moreover, mean annual health care expenditures are almost 30% higher among inactive adults com­pared to active adults [3]. There is a critical need for high-reach, cost-effective interventions to increase physical activity given the substantial public health burden and expenses related to such high levels of inactivity.

Internet-based interventions have great potential for widespread dissemination, thus many recent studies have focused on using this platform for physical activity promotion. In fact, a recent comprehensive review of Web-based physical activity interventions for adults identified 72 such programs, 44 of which (61%) reported significant increases in physical activity [4]. However, the review noted that this work was conducted in predominantly non-Latino, white samples and called for better representation of underserved populations, specifically racial and ethnic minorities, in future Internet-based physical activity intervention studies.

Latinos are the largest ethnic minority in the United States [5] and suffer marked health disparities. According to the US Census Bureau, Latinos (53 million) made up 17% of the US population growth between 2000 and 2010, accounting for more than half of the nation’s population growth [6]. Latinos report particularly low rates of physical activity and are disproportionately affected by related health conditions, especially Latina women. Only 38.2% of Latinas in the United States report meeting the federal guidelines for performance of aerobic physical activity (ie, >150 minutes/week of at least moderate-intensity aerobic activity), which is markedly less than non-Latina white women (50.9 %) and Latino men (47.0 %). Moreover, Latinas are more likely to be obese than non-Latina white women and experience excess burden from inactivity-related conditions, such as diabetes and stroke [7].

Evidence suggests that Internet-delivered interventions may be cost-effective. Past research in Dutch adults over 50 years of age indicated that Web-delivered interventions are a cost-effective way to increase physical activity when compared to no intervention [8]. Internet-delivered interventions may represent a particularly appropriate low-cost approach to physical activity promotion in Latinas due to the Internet’s ability to reach large numbers of people in the convenience of their own homes and address barriers to physical activity participation commonly cited by Latinas (eg, childcare and transportation) [9]. While in past years Internet use among Latinos was lower than among non-Latino whites, Internet use has grown markedly among Latinos in recent years, such that 81% of Latino adults report using the Internet compared to 85% of non-Latino white adults. Internet-based interventions, therefore, may be both low cost and have potential for broad dissemination in this population.

Our research team has developed a theory-based, individually tailored, Internet-delivered intervention [10,11] that was adapted for use in Latinas through extensive formative research—focus groups, cognitive interviews, and pilot studies—and tested in a randomized controlled trial [12]. Increases in minutes per week of at least moderate-intensity aerobic physical activity were significantly greater in the Internet intervention arm compared to the wellness contact control arm at 6 months [13] and were largely sustained at 12 months. However, to date there have been no cost or cost-effectiveness analyses conducted of such Internet-based physical activity interventions in Latinas. Therefore, the purpose of this paper is to assess the costs of this linguistically and culturally adapted Internet-based physical activity intervention for Latinas compared to the costs of a Web-based contact-matched control group. The purpose is also to evaluate the cost-effectiveness of this intervention for increasing moderate- to vigorous-intensity physical activity (MVPA) in Latinas.

## Methods

### Design

The Pasos Hacia La Salud study was a randomized controlled trial of an individually tailored, Spanish-language, Web-based physical activity intervention compared to a Web-based wellness control. Participants in the intervention condition group were given access to a website with individually tailored physical activity information; they also received monthly, personalized reports for 12 months. Participants in the control condition group were given access to a website with information on wellness topics other than physical activity (eg, diet and stress reduction); they also received alerts to access new materials with the same frequency as the intervention group. The primary outcome was weekly minutes of MVPA at 6 months measured by the 7-Day Physical Activity Recall (PAR) interview. Activity was also measured by accelerometer. A secondary outcome was minutes of MVPA at 12 months.

### Setting and Sample

Participants included 205 adult Latinas recruited from the community in San Diego County, California. Eligible participants were between 18 and 65 years of age, underactive (ie, engaging in less than 60 minutes per week of MVPA), and self-identified as Latina and/or Hispanic women. The study focused on women because our formative research showed that common barriers, motivators, and activity preferences were markedly different between Latino men and Latina women. In addition, physical inactivity is much more common among Latina women than among Latino men [14]. Exclusion criteria included current or planned pregnancy, plans to move from the area within 12 months, a body mass index of 45 kg/m^2^ or greater, and any health condition that might make unsupervised physical activity unsafe as determined by the Physical Activity Readiness Questionnaire [15], including history of heart disease, stroke, diabetes, or orthopedic problems. Participants also had to be willing to be randomized to either of the two conditions. A detailed breakdown of participant eligibility and flow diagram of participant allocation has been published elsewhere [12].

Human subjects research approval was granted by the University of California, San Diego, Human Research Protections Program, and all participants gave written informed consent.

### Intervention

The intervention was based on the Transtheoretical Model and Social Cognitive Theory. Participants filled out monthly online surveys about physical activity, cognitive and behavioral strategies to change behavior, self-efficacy, and other psychosocial constructs. Responses to these surveys were used to generate individually tailored reports for each participant, with feedback on changes over time and information about how their answers compared to those of other active women. Participants also received online physical activity manuals, which were matched to their readiness for changing physical activity behavior. Other features of the website included a calendar for goal setting and logging daily minutes of activity and steps, a message board for interacting with other participants, an *ask the expert* page, and a guide to local free and low-cost physical activity resources. Participants received regular emails with tip sheets on topics such as finding time to exercise, staying motivated, and other topics highlighted in formative research as being important to this population, such as childcare and cultural norms. An initial on-site visit was held to conduct a goal-setting session and orient participants to the website. At this initial visit, participants also received a pedometer for entering their daily steps on the website activity-tracking calendar, a binder with physical activity information sheets, a music CD, staff contact information, and the option of taking home a Spanish-language exercise DVD.

The first 6 months comprised the intensive intervention stage. Each month participants filled out questionnaires and received a tailored report and stage-matched online manual. Participants also received regular emails prompting them to access new information sheets and other materials on the website—weekly emails in month 1, biweekly emails in months 2 and 3, and monthly emails in months 4-6. All participants received a phone call after 1 week to help with pedometer and website use; received another call at 1 month to check in, answer questions, and help with goal setting; and participated in a repeat goal-setting session at 6 months. The second 6 months comprised a maintenance phase, during which participants received a monthly email prompt to visit the website to fill out the questionnaire to generate a personalized report. A final call to check in was made at month 9. The website and all materials were in Spanish.

### Wellness Contact Control

The control group received access to a Spanish-language website with a similar look and feel as the physical activity-based intervention site. However, this site included information on health topics other than physical activity, including diet, stress reduction, and sleep. In order to control for contact time, participants also engaged in an initial visit to be oriented to their website, received emails on the same schedule as the intervention group with new information sheets on various wellness topics, and filled out monthly questionnaires on wellness topics. Like those in the intervention condition group, they also received a phone call to check in at months 1 and 9, and had a short site visit at month 6.

### Measures

#### Clinical Outcome Measures

The primary outcome, upon which the study was powered, was change in MVPA from baseline to 6 months as measured by the 7-Day PAR. The 7-Day PAR is a self-report measure administered by trained, certified interviewers that asks participants to report the amount of weekly minutes spent in activities of different intensities—light, moderate, hard, very hard, and sleep—across a range of settings and activity types (eg, leisure, transport, and occupational). This measure has shown acceptable reliability and congruent validity with more objective physical activity measures and shows sensitivity to changes over time [16,17]. Staff members performing the 7-Day PAR were blinded to condition.

As an additional primary outcome, participants also wore ActiGraph GT3X+ accelerometers for the week corresponding to the self-report measure at the same three time points—baseline, 6 months, and 12 months. A minimum count of 1952 was set as a threshold for MVPA and a minimum bout duration of 10 minutes was used. Valid wear time was considered as at least 10 hours of wear on at least 5 days or at least 3000 minutes of wear time on at least 4 days.

#### Costs

Costs were estimated from a payer perspective and included all costs necessary to deliver the developed Web-based intervention in a clinical or community setting. This included staff time for training and delivering the intervention (ie, salary, benefits, and overhead) and cost of website maintenance and materials based on actual costs incurred during the trial. Costs associated exclusively with research activities, such as baseline and follow-up assessments, obtaining consent, and participant compensation, were not included. We also did not include costs of developing the website, as delivering the intervention would utilize the existing website and would not require further development. Costs associated with maintaining the website, such as Web hosting and technical support, were included.

#### Personnel Time

Costs for personnel were calculated by multiplying the time required for specific tasks—for the intervention and control groups separately—by standard University of California salary and fringe rates. Personnel time was determined by asking research staff to log the amount of time spent on nonresearch-related activities. This included training time for both the trainer and trainee; conducting initial baseline goal-setting visits and 6-month visits; scheduling and conducting 1-week, 1-month, and 9-month calls, including time for failed contact attempts; and time to compile study materials, such as the binder and CD.

Staff time also included routine maintenance of the websites, including checking the message board for appropriate comments and responding to *ask the expert* questions. It also included time needed to resend messages and materials, such as replacing lost pedometers or sending emails to corrected email addresses.

Staff costs were based on standard published salaries at the University of California, San Diego, for research staff qualified for each task. A masters- or PhD-level trainer’s annual salary was US $60,000 plus 44% benefits or US $86,520 annually (US $43.26 per hour) and a research associate with a bachelor’s degree received US $38,941 annual salary plus 44% benefits or US $56,153 annually (US $28.08 per hour). These hourly costs were increased by 10% to account for overhead costs of shared space.

#### Website Costs

Costs associated with using the website to deliver the intervention in a community or clinic setting included Web hosting and regular IT support. Web hosting was priced at US $75 per month for each website. Technical support was estimated at 2 hours per month at a standard rate of US $95 per hour. These costs were based on actual costs charged by the Web developer (Illumina Interactive) during the trial once the website was developed and being used by participants. Costs were equal for the intervention and control group websites.

#### Materials

Material costs were based directly on what was actually spent in the trial. Materials included the study binders, music CDs, labels, business cards, and paper and ink for tip sheets in the initial study binder. Costs for these came from standard wholesale office supply prices. Those in the intervention arm also received an Accusplit pedometer at a cost of US $12.50 each. A video library of approximately 15 DVDs was also available for participants to borrow from; the DVDs cost approximately US $10 each.

#### Hardware

Hardware costs included a standard desktop computer and a printer for printing tip sheets for the study binders. Hardware costs were estimated using market prices in June 2014; costs were depreciated using a straight-line method assuming a 5-year depreciation period and 3 years of use in the study. Hardware costs were equal for the intervention and control groups.

### Analysis

Costs were calculated as the total of all materials, hardware, personnel time including overhead and fringe benefits, website hosting, and website maintenance needed to run the study with the given number of participants. Research and development costs were not included. Change in total physical activity over the course of the study was calculated using linear interpolation of minutes from baseline to 6 months, then 6 months to 12 months, subtracting baseline physical activity. This was done for both subjective (7-Day PAR) and objective (accelerometer) measures of MVPA. Increase in physical activity was calculated using unadjusted mean weekly minutes at each time point for participants completing the trial (172/205, 83.9%). Dropout was equal across arms and there were no significant differences in any measures at baseline between those who did and did not complete the trial. There was also no significant difference between adjusted and unadjusted mean differences in MVPA. Cost-effectiveness was defined as the cost-per-minute increase in activity in each arm. This was determined by calculating the total cost per person at each time point, over the first 6 months and over the whole 12 months, and dividing this by the average total increase in physical activity across each time period. This was done separately for each condition in order to compare cost-effectiveness between the intervention and control groups. Finally, the incremental cost was defined as the additional cost-per-minute increase in the intervention condition beyond the change in MVPA in the control group; this was calculated by dividing the difference in change in MVPA between the two conditions by the difference in cost between the two conditions.

Sensitivity analyses were conducted by determining how cost-effectiveness, specifically incremental costs, would be impacted if personnel costs or intervention effectiveness increased or decreased by 20%. Because the cost per person could also vary depending on the number of participants enrolled, we also modeled how costs would change with varying participant numbers, multiplying the cost per person by the number of participants, but keeping fixed costs the same. Fixed costs included hardware (eg, computer and printer), technical support and Web hosting, and training costs. Variable costs included materials (eg, binders, paper, and pedometers) and the time for staff to deliver the intervention.

## Results

### Costs

Costs associated with running the intervention and control groups are presented in Table 1. Total cost of conducting the intervention at 6 months was US $10,712, which corresponded to a cost of US $17.17 per person per month. In contrast, the cost of running the control condition at 6 months was US $4900, or US $8.09 per person per month.

**Table 1:** Costs of study components for the intervention and control groups for the first 6 months (intensive intervention) and across the whole 12-month study period (N=205).

Item		Costs for intervention group (n=104), US $		Costs for wellness control group (n=101), US $	
		6 months	12 months (cumulative)	6 months	12 months (cumulative)
**Personnel**					
	Training	$471	$471	$157	$157
	Intervention delivery	$5674	$8083	$1976	$3119
**Website**					
	Maintenance	$270	$490	$8	$10
	Hosting	$450	$900	$450	$900
	Technical support	$1140	$2280	$1140	$2280
**Hardware**					
	Computer	$420	$420	$420	$420
	Printer	$240	$240	$240	$240
**Materials**					
	Pedometers	$1300	$1300	N/A^a^	N/A
	Paper, ink, binders, etc	$597	$597	$510	$510
	Videos	$150	$150	N/A	N/A
Total cost		$10,712	$14,781	$4900	$7634
Average cost per participant		$103	$142	$49	$76
Average cost per participant per month		$17	$12	$8	$6

^a^N/A: not applicable.

Cumulative total costs at 12 months were US $14,781 for the intervention group and US $7634 for the control group. Compared to costs at 6 months, this corresponded to a lower monthly cost of US $11.84 per participant per month for the intervention group and US $6.30 per participant per month for the control group. The largest expense for both conditions was personnel time, which was primarily devoted to conducting initial baseline visits, followed by IT support. The cost of providing pedometers in the intervention condition was also relatively high.

**Figure 1:**
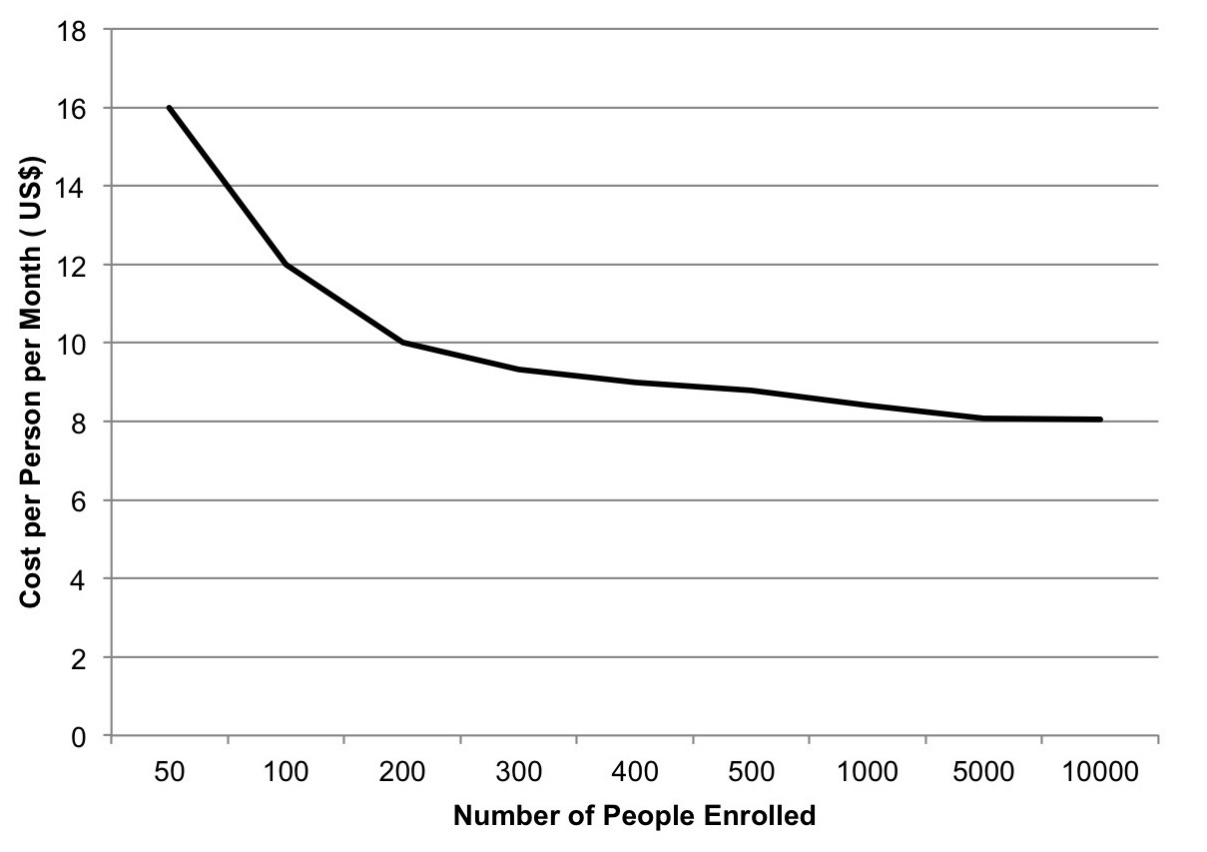
Cost per person per month over 12 months by number of participants enrolled.

Figure 1 shows the cost to deliver the intervention per person over the entire 12 months modeled by number of people enrolled. With only 50 people enrolled, the cost per person per month was US $16; this dropped to US $12 with 100 people and US $10 with 200 people. Costs dropped off more slowly with 300 people or more, reaching an asymptote near US $8 per person per month.

### Clinical Outcome Measures

As reported elsewhere [13], MVPA increased from baseline to 6 months in both conditions. This change was significantly greater in intervention group participants, who increased from an average of 8.0 (SD 15.0) minutes of self-reported MVPA per week at baseline to an average of 112.8 (SD 97.1) minutes at 6 months. Baseline minutes were similar in control group participants, which were an average of 8.5 (SD 14.6) minutes per week, but only increased to an average of 63.5 (SD 88.7) minutes per week at 6 months. Assuming a linear increase in MVPA across the first 6 months of the study, this corresponds to a total increase of 1362 minutes of MVPA per person in the intervention group versus 715 minutes of MVPA per person for the control group.

Changes recorded by objective measures were smaller, but still significantly greater in the intervention group than the control group. Intervention group participants increased from an average of 35.8 (SD 69.7) minutes per week at baseline to an average of 75.8 (SD 91.0) minutes per week at 6 months. Control group participants increased from an average of 28.7 (SD 48.2) minutes per week at baseline to an average of 43.0 (SD 60.9) minutes per week at 6 months. Using linear interpolation assuming a linear increase in minutes, this corresponds to a total increase of 523.0 minutes of MVPA per person in the intervention group versus 186.3 minutes of MVPA per person for the control group.

Increases in MVPA were largely sustained at 12 months. On average, intervention group participants reported an average of 108.6 (SD 107.2) weekly minutes of self-reported MVPA at 12 months compared to an average of 75.9 (SD 89.8) minutes in the control group. Assuming a linear change from baseline to 6 months and from 6 months to 12 months, this corresponds to a total increase of 4032.6 minutes per person in the intervention group over the course of the study and 2306.2 minutes per person in the control group. Weekly minutes recorded by accelerometers at 12 months were an average of 70.4 (SD 86.4) for the intervention group participants versus an average of 55.5 (SD 74.6) for control group participants. This corresponds to an increase of 1496 minutes per person over the course of the study for the intervention group and 695 minutes per person for the control group.

### Cost-Effectiveness

Table 2 Table 2 provides the estimates of cost-per-minute increases in MVPA in the intervention and control arms. Over 6 months, costs for the intervention arm were US $0.08 per minute increase per person for self-reported minutes and US $0.20 per minute per person for accelerometer-recorded minutes of MVPA. For the control group, these costs were US $0.07 and US $0.26 per minute increase per person, respectively. Costs per minute decreased for both arms over the whole 12 months: after 1 year, the intervention group costs were US $0.04 per minute increase per person for self-reported minutes and US $0.10 per minute increase per person for accelerometer-measured minutes of MVPA. For the control group, these costs were US $0.03 and US $0.11 per minute increase per person, respectively.

**Table 2:** Cost-per-minute increases in physical activity during the initial 6-month intervention and the full 12-month study (cumulative).

		Intervention group		Wellness control group	
		7-Day Physical Activity Recall	ActiGraph accelerometer	7-Day Physical Activity Recall	ActiGraph accelerometer
**Total MVPA^a^** **minutes per person, minutes**					
	Baseline to 6 months	1362	523	715	186
	Total 12 months	4033	1496	2306	696
**MVPA minutes per person per month, minutes**					
	Baseline to 6 months	227	87	119	31
	Total 12 months	336	125	192	58
**Cost-per-minute increase in MVPA, US $**					
	Baseline to 6 months	$0.08	$0.20	$0.07	$0.26
	Total 12 months	$0.04	$0.10	$0.03	$0.11
**Incremental cost-per-minute increase in MVPA, US $**					
	Baseline to 6 months	$0.08	$0.16	N/A^b^	N/A
	Total 12 months	$0.04	$0.08	N/A	N/A

^a^MVPA: moderate- to vigorous-intensity physical activity.

^b^N/A: not applicable.

Incremental costs for increases in MVPA (ie, cost-per-minute increases in the intervention group beyond that seen in the control group) varied by time point and measurement method. At 6 months, incremental costs were US $0.08 per minute for the 7-Day PAR and US $0.16 per minute for accelerometer-measured MVPA. At 12 months, these were US $0.04 per minute and US $0.08 per minute, respectively.

Sensitivity analyses (see Table 3) examined how changes in staffing costs and intervention effectiveness would influence cost-effectiveness, specifically incremental costs. A 20% increase in staffing costs yielded an increased incremental cost at 6 months from US $0.08 to US $0.10 per minute—US $0.16 to US $0.19 for accelerometers—while a 20% decrease in staffing costs yielded a decrease to US $0.07 and US $0.014 per minute for subjective and accelerometer-measured minutes, respectively. Variations in intervention effectiveness (ie, changes in MVPA minutes) yielded nearly identical changes. For all sensitivity analyses, changes in incremental costs were quite small at 12 months.

**Table 3:** Sensitivity analyses for incremental cost-per-minute increases in moderate- to vigorous-intensity physical activity in the intervention versus control groups.

Cost and effectiveness measures		6-month incremental cost per minute, US $		12-month incremental cost per minute, US $	
		7-Day Physical Activity Recall	ActiGraph accelerometer	7-Day Physical Activity Recall	ActiGraph accelerometer
Standard calculation		$0.08	$0.16	$0.04	$0.08
**Staffing costs**					
	+20%	$0.10	$0.19	$0.04	$0.10
	-20%	$0.07	$0.14	$0.03	$0.07
**Intervention effectiveness**					
	+20%	$0.07	$0.13	$0.03	$0.07
	-20%	$0.11	$0.20	$0.05	$0.12

## Discussion

### Principal Findings

Our Internet-based physical activity intervention for Spanish-speaking Latinas, which was previously shown to be effective for increasing MVPA, was also low cost with a cost of just US $12 per person per month over the whole 12-month period. One of the key benefits of an Internet-based intervention is the ability to scale the intervention for larger samples. Results showed that the cost per participant in the intervention group could be reduced from US $12 down to US $8 per month, with only about 300 users in the intervention arm. The most expensive cost by far was personnel time. Interventions that utilize automated systems that require little to no staff time are important for keeping the cost of physical activity programs low. This study utilized an automated system while still personalizing intervention materials. The greater cost of running the intervention condition was primarily driven by the increased staff time required to orient participants to the website, perform an individual goal-setting tutorial, and make check-in calls.

Although the monthly cost of the intervention arm was more expensive than the control arm, participants in the intervention arm increased their physical activity significantly more than those in the control arm. Consequently, the cost per minute increase was similar between the two groups, with the control arm being slightly more cost-effective for subjectively measured minutes and the intervention arm being slightly more cost-effective for objectively measured minutes. These results suggest that this Web-based intervention could produce significantly greater increases in MVPA at a similar price per minute. Given the vast benefits of physical activity and the low incremental cost of just US $0.04 per minute beyond that seen in the control arm, this Web-based intervention is a cost-effective approach to health promotion.

### Comparison With Prior Work

TThe costs of this Web-based intervention are considerably lower than a printed mail-delivered version of this program completed with Latina women, which cost US $17 per person per month [18]. The mail-delivered intervention was slightly less effective and more costly, thus the cost per minute at 6 months was also significantly higher in the mail-delivered program (US $0.18) than in the Web-based program (US $0.08). These costs would likely diverge farther with wider dissemination, as many of the costs of the Internet intervention do not increase with increased users, while the print version requires postage, printing materials, mailing materials, and staff time to process tailored questionnaires for each person.

It is difficult to compare these costs to other Web-based interventions, as few Internet-based physical activity interventions have examined cost-effectiveness and none could be found that examined it with underserved populations. Additionally, across cost-effectiveness studies there is a lack of homogeneity regarding which variables are included when calculating costs, preventing direct comparisons between studies. For example, one paper comparing two Web-based studies reported much lower average costs for conducting their interventions than this study; however, no staff time was included [8]. In addition, while some analyses included recruitment costs, we did not include them in this study as recruitment costs incurred during the trial were for a research study and were therefore not representative of recruitment costs for delivering the intervention in a real-world setting. Some recruitment costs would likely be necessary in a clinical or community setting, though these would vary greatly depending on whether they targeted an existing patient or membership base or the community at large.

On that note, costs reported here should be seen as a guide to potential delivery costs rather than an exact estimate, as costs would likely vary widely depending on delivery site. The intervention examined here could potentially be implemented in a health system or community center, each of which would have vastly different costs for staffing, recruitment (if any), and overhead. Sensitivity analyses showed that cost-effectiveness, measured by incremental costs, would remain relatively stable despite ranges in staffing costs. Larger variations were seen in cost-effectiveness when examining change in intervention effectiveness, which would likely range greatly across populations. Variations in cost-effectiveness were perhaps modeled best in this study by comparing subjective and objective measures of physical activity, which showed marked differences in activity gains. These differences in reported minutes could be due to subjective reporting bias or inaccurate recall of duration or intensity of activities. Some discrepancy between them should be expected, however, as accelerometry and the 7-Day PAR measure different behaviors (ie, activity vs hip acceleration). Accelerometry is also unable to accurately measure some activities, such as biking and swimming, and underestimates energy expenditure for some activities, such as hiking and walking on an incline or activities using upper body movement [19].

As the purpose of this study was to estimate the cost of delivering the already-developed intervention, we did not take into account the cost associated with developing the intervention. A previous study with an Internet-delivered intervention for adults did examine the development costs [20] and determined that although developing the intervention cost about US $100,000, only 352 participants would be needed to overcome the start-up costs of the Internet intervention relative to delivering the print-based version. Since start-up costs only occur once and may be easily mitigated, our focus on cost of dissemination is important as some costs, such as pedometers, do not decrease with an increased number of participants.

### Strengths and Limitations

One limitation is that we did not consider costs for updating the website over time. As technology grows and changes it is important to have a website that changes over time as well, which may incur costs over that of regular maintenance and technical support. Over the 3 years of running this intervention, the main modifications to the website were updating resources, which study staff were able to do; we were not able to estimate changes that would be of significant cost to updating the website in future years. The Web-based contact control group allowed for an assessment of intervention effectiveness, but did not allow for a comparison of costs across delivery modalities. Additionally, this analysis estimated costs from the payer perspective, but was unable to estimate costs from a societal perspective. We also did not include recruitment costs, as those associated with the trial would not be a good estimate of recruitment costs in a nonresearch setting.

This study has a number of strengths, including using data from a randomized controlled trial and relying on current market data for materials, equipment, and personnel costs. This study also targeted Spanish-speaking Latinas and demonstrated cost-effectiveness at the end of the intervention (ie, 6 months) and during the maintenance phase (ie, 12 months). Additionally, cost-effectiveness was calculated using both self-reported and objectively measured physical activity.

Given the disparities in chronic diseases among Latinas, next steps should be to determine whether physical activity interventions could reduce health care costs. This study focused on relatively short-term physical activity outcomes and did not collect information on health care costs. Future research that includes health care costs could provide a more comprehensive understanding of the potential costs and quality-adjusted life years that could be gained from increasing physical activity in Latinas [21]. As health habits in Latino families are more closely related than in non-Latino white families, particularly among mothers and children [22], future research should also investigate how increasing physical activity in Latina women could influence health and health care costs for their families and communities.

### Conclusions

Results from this study indicated that a tailored, Internet-delivered intervention is a cost-effective approach to increasing physical activity among Latinas that has the potential for dissemination. Using the Internet allowed for the delivery cost of the intervention to be cut in half as the number of participants using the intervention increased. Large-scale implementations of interventions that demonstrate cost-effectiveness have the potential to reduce health disparities, benefiting public health.

## Abbreviations

MVPA: moderate- to vigorous-intensity physical activity
N/A: not applicable
PAR: Physical Activity Recall
